# Broadband Sound Insulation and Dual Equivalent Negative Properties of Acoustic Metamaterial with Distributed Piezoelectric Resonators

**DOI:** 10.3390/ma15144907

**Published:** 2022-07-14

**Authors:** Zhifu Zhang, Jiaxuan Wang, Zhuang Li, Xirui Zhang

**Affiliations:** 1 Mechanical and Electrical Engineering College, Hainan University, Haikou 570228, China; jeff.zfzhang@foxmail.com; 2 State Key Laboratory of Digital Manufacturing Equipment and Technology, Huazhong University of Science and Technology, Wuhan 430074, China; chiahsuan_wang@foxmail.com (J.W.); lz_mse@hust.edu.cn (Z.L.)

**Keywords:** sound insulation, acoustic metamaterial, negative property, piezoelectric array, resonant shunt, subwavelength structure

## Abstract

Aiming at the unsatisfactory sound transmission loss (STL) of thin-plate structures in the low-mid frequency range, this paper proposes an acoustic insulation metamaterial with distributed piezoelectric resonators. A complete acoustic prediction model is established based on the effective medium method and classical plate theory, and the correctness is verified by the STL simulation results of the corresponding acoustic-structure fully coupled finite-element model. Moreover, the intrinsic relationship between the dual equivalent negative properties and STLs is investigated to reveal the insulation mechanisms of this metamaterial. Then, the influence of the geometric and material parameters on the double equivalent negative characteristics is studied to explore the broadband STL for distributed multi-modal resonant energy-dissipation modes in the frequency band of interest. The results show that the two acoustic insulation crests correspond to the dual equivalent negative performances, and the sound insulation in the low-mid frequency range is improved by more than 5 dB compared with that of the substrate, even up to 44.49 dB.

## 1. Introduction

Noise pollution is one of the most serious environmental problems, and pollution levels continue to worsen as a result of modern industries and transportation [1,2,3]. Conventional materials are unquestionable in meeting the functional requirements of equipment and facilities, but they are stretched when taking into account the need for lightweight and quiet. For this reason, acoustic metamaterials with negative properties have been favored by researchers in recent years. The concept of negative characteristics was firstly proposed in the electromagnetic field by Veselago [4], who postulated a theory for possible materials having negative electric permittivity and magnetic permeability, hence resulting in a negative refractive index. However, the actual focus began in 2000, when Smith [5] demonstrated a composite medium that exhibits a frequency region in the microwave regime with simultaneously negative values of effective permeability and permittivity. Since then, artificially constructed metamaterials have achieved unprecedented development in the fields of electromagnetics [6,7,8], optics [9,10,11], mechanics [12,13,14], and acoustics [15,16,17].

Based on the mathematical analogy between acoustic and electromagnetic waves, acoustic metamaterials with local resonant energy-dissipation modes have sprung up, and the common local resonance structures include core-coated concentric microstructures [18,19], mass-spring resonators [20,21], Helmholtz resonators [22,23], reverse vibration of adjacent cells [24,25], and resonant piezoelectric shunts [26,27]. Among them, piezoelectric resonators, with the advantages of small additional mass [28], no need for control energy [29], controllable shunt damping [27], and considerable potential [30], have promoted further research on acoustic metamaterials containing periodic piezoelectric patches for vibration and noise control.

In particular, Hollkamp [31] experimentally explored the multi-modal suppression of a periodic structure by attaching multiple pairs of peizoceramics containing shunt dampers to an aluminum beam. Thorp et al. [29] developed a mathematical model to investigate the longitudinal wave attenuation characteristics of rods with periodic shunted piezoelectric patches and found that the resulting periodic structure can block wave propagations in specific frequency bands, while the position and width of stopbands are adjustable. Then, Thorp et al. [30] extended the research on the wave propagation performance of fluid-loaded shells with periodic shunted piezoelectric rings, and the results showed that the suitable shunt strategy can generate an additional stopband at the tuning frequency. Casadei et al. [26] designed a periodic 4 × 4 layout of RL shunted piezoelectric patches, to achieve broadband vibration reduction in a flexible isotropic plate in tunable frequency bands. Moreover, Casadei et al. [27] applied the periodic piezoelectric array extension to a flexible plate in a closed cavity and investigated the attenuation properties of broadband acoustic radiation by numerical and experimental methods. Airoldi and Ruzzene [32] presented a one-dimensional metamaterial consisting of periodic shunted piezoelectric patches and a homogeneous beam with the aid of the transfer matrix method to predict the occurrence of bandgaps at the tuning frequencies and estimate wave attenuations. Casadei et al. [33] proposed a concept of a tunable acoustic waveguide equipped with periodic piezoelectric resonator arrays on a phononic crystal plate containing cylindrical stubs to realize the excellent characteristics of negative group velocity at a specific frequency. Jin et al. [34] designed a periodic acoustic metamaterial with piezoelectric resonator structures based on the unit cell of piezoelectric composites encasing a hard-core and obtained the unconventional characteristics of negative effective mass density and elastic modulus. Hou and Assouar [35] constructed a one-dimensional layer-stacked acoustic metamaterial from non-piezoelectric and piezoelectric materials with LC circuits and predicted that the layer-stacked system has a negative elastic modulus by the transfer matrix method.

Although there are numerous studies on the vibroacoustic property of acoustic metamaterials containing periodic piezoelectric arrays, few have focused on sound transmission loss (STL). For example, Qi et al. [36] proposed a planar acoustic metamaterial consisting of an acoustic energy confinement part and strain energy conversion module for energy harvesting and analyzed the STL in the frequency band of 1.8–2.5 kHz based on COMSOL. Zhang et al. [28] pasted square piezoelectric patches with resonant shunts on a substrate to form a two-dimensional metamaterial and investigated the equivalent material properties and STL by means of effective medium (EM), and then found that the metamaterial has an equivalent negative dynamic bending stiffness and the corresponding sound insulation peak. Kaijun et al. [37] adopted a homogenization method to explore the STL within 8 kHz for a piezo-electromechanical plate with similar periodic structures. Furthermore, Zhang et al. [38] developed an acoustic prediction model for orthogonal rib-stiffened sandwich structures with periodic piezoelectric arrays, by integrating the EM method, Kirchhoff’s thin plate theory, and virtual work principle, in order to investigate the low-frequency broadband STL characteristics.

However, the existing studies on acoustic insulation metamaterials have two main shortcomings: (1) They mainly focus on single-negative metamaterials, and there are few studies on double-negative metamaterials. (2) Most of them focus on the goal of acoustic characteristics, ignoring the intrinsic relationship between equivalent negative properties and STLs. Therefore, in this paper, an acoustic insulation metamaterial with distributed piezoelectric resonators is designed, which is formed by flexibly connecting periodic subwavelength piezoelectric arrays with shunt circuits to a homogeneous substrate. For the purpose of exploring the STLs and dual equivalent negative properties, the following three aspects of work are mainly carried out. The first is to design a new acoustic insulation metamaterial and develop an acoustic theoretical prediction model based on the EM method and classical plate theory (CPT) [39]. The next one is to establish the acoustic-structure fully coupled finite-element model of the present metamaterial with the help of COMSOL Multiphysics and perform the simulation verification of STL. Last but not least is to reveal the sound insulation mechanisms and investigate the relationship between the double equivalent negative characteristics and STLs, with a view to delving into the broadband STL in the low-mid frequency range.

## 2. Theoretical Modeling and Methodology

### 2.1. Physical Model

**Figure 1:**
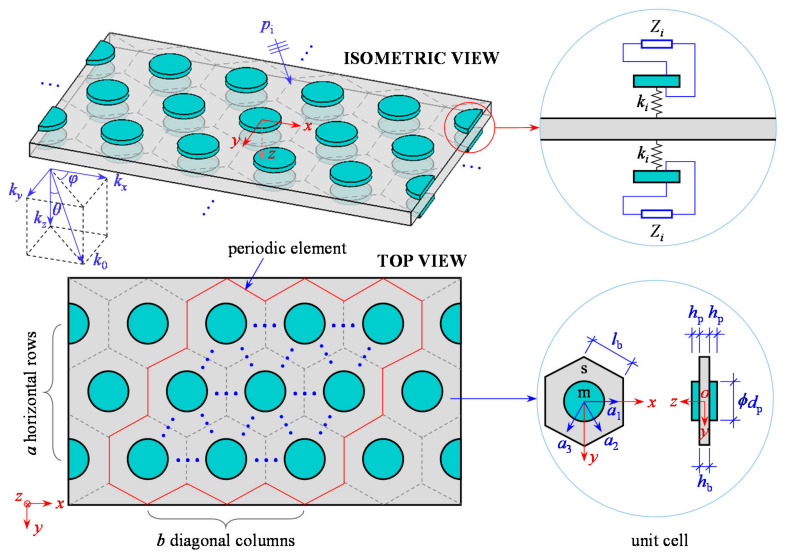
Schematic diagram of the metamaterial thin-plate containing subwavelength piezoelectric arrays with resonant shunts.

The acoustic insulation metamaterial thin-plate is composed of an infinite substrate with a thickness of *h*_b_ as the main structure, and the periodic subwavelength piezoceramic patches are symmetrical to the upper and lower sides, which presents a two-dimensional periodic staggered arrangement of regular hexagonal prism unit cells globally, as shown in the isometric view in Figure 1. Meanwhile, the parallelogram region of the red solid wireframe in the top view is a single periodic element and has a layout of *a* horizontal rows and *b* diagonal columns. Among them, each piezoelectric patch with diameter *d*_p_ and thickness *h*_p_ is externally connected with a shunt circuit with an electric impedance of *Z_i_* (*i* ∈ [1, *a*·*b*]), and the upper and lower two patches are flexibly connected to the substrate in pairs, forming a mass-spring system with complex stiffness *k_i_*. Moreover, a global Cartesian coordinate system is established on the upper surface of the substrate, and the directions of *x*- and *z*-axes are, respectively, along with the horizontal array of a unit cell and vertically downward, while the *y*-axis is perpendicular to the *xz*-plane forward.

A plane wave *p*_i_ with amplitude *P*_i_ impinging on the upper surface of the metamaterial thin-plate at elevation angle *θ* and azimuth angle *φ*, which can be expressed as:

(1) $$p_{i}\left(x,y,z\right)=P_{i}e^{-i\left(k_{x}x+k_{y}y+k_{z}z\right)}$$

with

(2) $$k_{x}=k_{0}\sin\theta \;\cos\varphi ,\;k_{y}=k_{0}\sin\theta \;\sin\varphi ,\;k_{z}=k_{0}\cos\theta ,\;k_{0}=\omega /c_{0}$$

where *c*_0_ is the sound speed of the service medium, and the time-dependent factor e^iωt^ is omitted in Formula (1).

It is worth noting that a unit cell in one periodic element shown in Figure 1 has periodicity in the directions ${\vec{a}}_{1}$, ${\vec{a}}_{2}$, and ${\vec{a}}_{3}$, which satisfies the following relationship [40]:

(3) $$k_{x}\vec{i}+k_{y}\vec{j}=n_{1}{\vec{a}}_{1}+n_{2}{\vec{a}}_{2}+n_{3}{\vec{a}}_{3}$$

### 2.2. Dual Equivalent Properties

**Figure 2:**
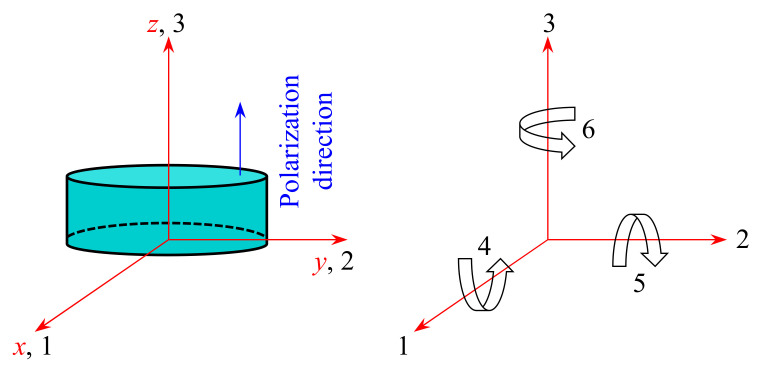
Schematic diagram of crystallographic axes and deformation directions.

For a unit cell in one single periodic element as displayed in Figure 1, the piezoelectric constitutive relations of transversely isotropic piezoceramics whose electropolar surfaces are vertical to the *z*-axis can be written in matrix form as [41]:

(4) $$\left\{\begin{array}{c} S_{1,i}\\ S_{2,i}\\ S_{3,i}\\ S_{4,i}\\ S_{5,i}\\ S_{6,i} \end{array}\right\}=\left[\begin{array}{cccccc} s_{11,i}^{E} & s_{12,i}^{E} & s_{13,i}^{E} & 0 & 0 & 0\\ s_{12,i}^{E} & s_{11,i}^{E} & s_{13,i}^{E} & 0 & 0 & 0\\ s_{13,i}^{E} & s_{13,i}^{E} & s_{33,i}^{E} & 0 & 0 & 0\\ 0 & 0 & 0 & s_{44,i}^{E} & 0 & 0\\ 0 & 0 & 0 & 0 & s_{44,i}^{E} & 0\\ 0 & 0 & 0 & 0 & 0 & 2\left(s_{11,i}^{E}-s_{12,i}^{E}\right) \end{array}\right]\left\{\begin{array}{c} T_{1,i}\\ T_{2,i}\\ T_{3,i}\\ T_{4,i}\\ T_{5,i}\\ T_{6,i} \end{array}\right\}+\left[\begin{array}{ccc} 0 & 0 & d_{31,i}\\ 0 & 0 & d_{31,i}\\ 0 & 0 & d_{33,i}\\ 0 & d_{15,i} & 0\\ d_{15,i} & 0 & 0\\ 0 & 0 & 0 \end{array}\right]\left\{\begin{array}{c} E_{1,i}\\ E_{2,i}\\ E_{3,i} \end{array}\right\}$$

(5) $$\left\{\begin{array}{c} D_{1,i}\\ D_{2,i}\\ D_{3,i} \end{array}\right\}=\left[\begin{array}{cccccc} 0 & 0 & 0 & 0 & d_{15,i} & 0\\ 0 & 0 & 0 & d_{15,i} & 0 & 0\\ d_{31,i} & d_{31,i} & d_{33,i} & 0 & 0 & 0 \end{array}\right]\left\{\begin{array}{c} T_{1,i}\\ T_{2,i}\\ T_{3,i}\\ T_{4,i}\\ T_{5,i}\\ T_{6,i} \end{array}\right\}+\left[\begin{array}{ccc} \varepsilon_{11,i}^{T} & 0 & 0\\ 0 & \varepsilon_{11,i}^{T} & 0\\ 0 & 0 & \varepsilon_{33,i}^{T} \end{array}\right]\left\{\begin{array}{c} E_{1,i}\\ E_{2,i}\\ E_{3,i} \end{array}\right\}$$

where [*s*^E^], [*d*] and [*ε*^T^] are, respectively, the elastic constant, piezoelectric strain constant, and dielectric constant. In the meantime, {*S*}, {*D*}, {*T*}, and {*E*} all represent tensors associated with piezoelectric patches, in order of strain, electric displacement, stress, and electric field strength. The meanings of subscripts in constants [∙] are illustrated in Figure 2, in which 1–3 imply normal strain/stress directions, while 4–6 denote shear strain/stress directions. However, for the tensors {∙}, the first and second subscripts indicate the direction of electric field and force, respectively.

Based on the plane stress state assumption [28,42], the piezoelectric constitutive equations are reformulated as follows:

(6) $$\left\{\begin{array}{c} S_{1,i}\\ S_{2,i}\\ S_{6,i}\\ D_{3,i} \end{array}\right\}=\left[\begin{array}{cccc} S_{11,i}^{E} & S_{12,i}^{E} & 0 & d_{31,i}\\ S_{12,i}^{E} & S_{11,i}^{E} & 0 & d_{31,i}\\ 0 & 0 & 2\left(S_{11,i}^{E}-S_{12,i}^{E}\right) & 0\\ d_{31,i} & d_{31,i} & 0 & \varepsilon_{33,i}^{T} \end{array}\right]\left\{\begin{array}{c} T_{1,i}\\ T_{2,i}\\ T_{6,i}\\ E_{3,i} \end{array}\right\}$$

Given that the electrical displacement *D*_3,*i*_ on the electrode of each piezoceramic patch remains invariable, the electric current generated is [43]:

(7) $$I_{i}=\frac{E_{3,i}h_{p,i}}{Z_{i}}=-A_{p,i}D_{3,i}s$$

where *s* = i*ω* is the Laplace operator, and $A_{p,i}=\pi /4\cdot d_{p,i}^{2}$ is the area of the piezoceramic electrode.

Combining Equations (6) and (7), there are

(8) $$E_{3,i}=-\frac{sZ_{i}C_{p,i}^{T}d_{31,i}\left(T_{1,i}+T_{2,i}\right)}{\varepsilon_{33,i}^{T}\left(1+sZ_{i}C_{p,i}^{T}\right)}$$

Here, $C_{p,i}^{T}$ is the intrinsic capacitance of the *i*-th shunted piezoelectric patch in the constant stress state, that is

(9) $$C_{p,i}^{T}=\frac{A_{p,i}\varepsilon_{33,i}^{T}}{h_{p,i}}$$

Substituting Equation (8) back into Equation (6) has

(10) $$\left\{\begin{array}{c} S_{1,i}\\ S_{2,i}\\ S_{6,i} \end{array}\right\}=\left[\begin{array}{ccc} s_{11,i}^{E}-\frac{sZ_{i}C_{p,i}^{T}d_{31,i}^{2}}{\varepsilon_{33,i}^{T}\left(1+sZ_{i}C_{p,i}^{T}\right)} & s_{12,i}^{E}-\frac{sZ_{i}C_{p,i}^{T}d_{31,i}^{2}}{\varepsilon_{33,i}^{T}\left(1+sZ_{i}C_{p,i}^{T}\right)} & 0\\ s_{12,i}^{E}-\frac{sZ_{i}C_{p,i}^{T}d_{31,i}^{2}}{\varepsilon_{33,i}^{T}\left(1+sZ_{i}C_{p,i}^{T}\right)} & s_{11,i}^{E}-\frac{sZ_{i}C_{p,i}^{T}d_{31,i}^{2}}{\varepsilon_{33,i}^{T}\left(1+sZ_{i}C_{p,i}^{T}\right)} & 0\\ 0 & 0 & 2\left(s_{11,i}^{E}-s_{12,i}^{E}\right) \end{array}\right]\left\{\begin{array}{c} T_{1,i}\\ T_{2,i}\\ T_{6,i} \end{array}\right\}$$

However, with regard to linear isotropic materials, the corresponding constitutive strain-stress relations are expressed by the following expressions, namely

(11) $$\left\{\begin{array}{c} S_{1}\\ S_{2}\\ S_{6} \end{array}\right\}=\frac{1}{E}\left[\begin{array}{ccc} 1 & -\upsilon & 0\\ -\upsilon & 1 & 0\\ 0 & 0 & 2\left(1+\upsilon \right) \end{array}\right]\left\{\begin{array}{c} T_{1}\\ T_{2}\\ T_{6} \end{array}\right\}$$

By comparing the Equations (10) and (11), each piezoceramic patch with an external shunt circuit can be equivalent to a linear isotropic material with Young’s modulus *E*_p,*i*_ and Poisson’s ratio *υ*_p,*i*_, respectively, as follows:

(12) $$E_{p,i}=\frac{\varepsilon_{33}^{T}\left(1+sZ_{i}C_{p,i}^{T}\right)}{s_{11,i}^{E}\varepsilon_{33}^{T}\left(1+sZ_{i}C_{p,i}^{T}\right)-sZ_{i}C_{p,i}^{T}d_{31,i}^{2}}$$

(13) $$\upsilon_{p,i}=-\frac{s_{12,i}^{E}\varepsilon_{33,i}^{T}\left(1+sZ_{i}C_{p,i}^{T}\right)-sZ_{i}C_{p,i}^{T}d_{31,i}^{2}}{s_{11,i}^{E}\varepsilon_{33,i}^{T}\left(1+sZ_{i}C_{p,i}^{T}\right)-sZ_{i}C_{p,i}^{T}d_{31,i}^{2}}$$

Moreover, as regards a single two-spring vibration system (one unit cell), assuming that the displacement of each segment in an equivalent spring is linearly distributed, and the equivalent volume density of the upper or lower vibrators in *i*-th mass-spring system is expressed as:

(14) $$\rho_{r,i}=\frac{\rho_{p,i}}{1-\frac{\omega^{2}}{\omega_{r,i}^{2}\left(1+i\eta_{r,i}\right)}}$$

Hence, on the basis of the classical laminated plate theory [44], the expressions for the surface density (SD) and bending stiffness (BS) of the single-layer region s and multi-layer region m in one periodic element as shown in Figure 1 can be derived in the following order.

(15) $$\sigma \left(x,y\right)=\left\{\begin{array}{ll} \sigma_{m}=\rho_{b}h_{b}+\frac{2}{ab}\sum_{i=1}^{ab}\rho_{r,i}h_{p,i}, & \left(x,y\right)\in m\\ \sigma_{s}=\rho_{b}h_{b}, & \left(x,y\right)\in s \end{array}\right.$$

(16) $$D\left(x,y\right)=\left\{\begin{array}{ll} D_{m}=\frac{E_{b}h_{b}^{3}}{12\left(1-\upsilon_{b}^{2}\right)}+\frac{1}{ab}\sum_{i=1}^{ab}\frac{E_{p,i}\left[{\left(h_{b}+2h_{p,i}\right)}^{3}-h_{b}^{3}\right]}{12\left(1-\upsilon_{p,i}^{2}\right)}, & \left(x,y\right)\in m\\ D_{s}=\frac{E_{b}h_{b}^{3}}{12\left(1-\upsilon_{b}^{2}\right)}, & \left(x,y\right)\in s \end{array}\right.$$

where *ρ*_b_, *E*_b_, and *υ*_b_ are the density, Young’s modulus, and Poisson’s ratio, respectively.

Therefore, given the subwavelength assumption [28,38,45], the acoustic metamaterial can be regarded as a homogeneous material when the periodic dimension is smaller than the flexural wavelength of the substrate. The dual equivalent properties of the metamaterial thin-plate are obtained according to the EM theory [28,32,45], as bellows:

(17) $$\sigma_{\mathrm{eq}}=\chi \sigma_{m}+\left(1-\chi \right)\sigma_{s}$$

(18) $$D_{\mathrm{eq}}=\frac{D_{m}D_{s}}{\left(1-\chi \right)D_{m}+\chi D_{s}}$$

where $\chi =\pi d_{p}^{2}/\left(6\sqrt{3}l_{b}^{2}\right)$ represents the area ratio of region m to region s in a unit cell.

### 2.3. Sound Transmission Loss Characteristics

The metamaterial thin-plate produces a simple harmonic vibration under the excitation of plane wave *p*_i_, which generates a reflected wave *p*_r_ and a transmitted wave *p*_t_ in the semi-infinite space on both sides of the incident and transmission ends, respectively. Thus, the *p*_r_, *p*_t_, and transverse displacement *w* can be expressed by [28,38,46]

(19) $$p_{r}\left(x,y,z\right)=P_{r}e^{-i\left(k_{x}x+k_{y}y-k_{z}z\right)}$$

(20) $$p_{t}\left(x,y,z\right)=P_{t}e^{-i\left(k_{x}x+k_{y}y+k_{z}z\right)}$$

(21) $$w\left(x,y\right)=We^{-i\left(k_{x}x+k_{y}y\right)}$$

Then, the governing equation for the acoustic metamaterial thin-plate as depicted in Figure 1 can be written by means of CPT [39] as

(22) $$D_{\mathrm{eq}}\nabla^{4}w\left(x,y\right)-\sigma_{\mathrm{eq}}\omega^{2}w\left(x,y\right)={\left.p_{i}\left(x,y,z\right)\right|}_{z=0}+{\left.p_{r}\left(x,y,z\right)\right|}_{z=0}-{\left.p_{t}\left(x,y,z\right)\right|}_{z=h_{b}}$$

where ∇^4^ = (*∂*^2^/*∂x*^2^ + *∂*^2^/*∂y*^2^)^2^.

The continuity condition at acoustic-structure interfaces is that the normal vibration velocity of metamaterial thin-plate is equal to the particle vibration velocity in fluid media, and the following relationships are obtained based on the momentum equation:

(23) $${\left.\frac{\partial \left[p_{i}\left(x,y,z\right)+p_{r}\left(x,y,z\right)\right]}{\partial z}\right|}_{z=0}=\rho_{0}\omega^{2}w\left(x,y\right)$$

(24) $${\left.\frac{\partial \left[p_{t}\left(x,y,z\right)\right]}{\partial z}\right|}_{z=h_{b}}=\rho_{0}\omega^{2}w\left(x,y\right)$$

Combining Equations (1) and (22)–(24), the amplitude coefficients of *p*_r_, *p*_t_, and *w* can be separated as follows:

(25) $$W=\frac{2P_{i}}{Z_{M}+2Z_{R}}$$

(26) $$P_{r}=\frac{Z_{M}}{Z_{M}+2Z_{R}}P_{i}$$

(27) $$P_{t}=\frac{2Z_{R}}{Z_{M}+2Z_{R}}P_{i}e^{ik_{z}h_{b}}$$

where *Z*_M_ and *Z*_R_ represent the mechanical and radiation impedances of the metamaterial thin-plate, respectively, and are expressed in turn as

(28) $$Z_{M}=D_{\mathrm{eq}}{\left(k_{x}^{2}+k_{y}^{2}\right)}^{2}-\sigma_{\mathrm{eq}}\omega^{2}$$

(29) $$Z_{R}=\frac{i\rho_{0}\omega^{2}}{k_{z}}$$

Therefore, the transmission coefficient and STL of the acoustic metamaterial excited by oblique incident plane waves are calculated by

(30) $$\tau =\frac{2Z_{R}}{Z_{M}+2Z_{R}}e^{ik_{z}h_{b}}$$

(31) $$\mathrm{STL}=-20\log_{10}\left|\tau \right|$$

## 3. Model Validation

For the acoustic metamaterial with subwavelength piezoelectric arrays shown in Figure 1, the piezoceramic and substrate materials are PZT-5H [38,47] and aluminum (Al) [45,48], respectively, and the corresponding geometric properties are, in order, *l*_b_ = 10 mm, *h*_b_ = 2 mm, *d*_p_ = 10 mm, and *h*_p_ = 1 mm. Given that the maximum computational frequency of the metamaterial is limited by the subwavelength assumption [28,38,45], it requires the minimum linearity (*l*_min_) of a single unit cell is smaller than the flexural wavelength (*λ*_b_) of the substrate, namely, 0 < *l*_min_/*λ*_b_ < 1, and there is

(32) $$\frac{l_{\min}}{\lambda_{b}}=\frac{\sqrt{3}}{2\pi }l_{b}{\left(\frac{D_{s}}{\sigma_{s}\omega^{2}}\right)}^{-1/4}$$

Taking *l*_min_/*λ*_b_ = 0.5 in this work, the theoretical highest calculation frequency is *f*_max_ = 16.3 kHz. Therefore, the subwavelength hypothesis is valid in the frequency range below *f*_max_, and the EM method is accurate and reliable.

**Figure 3:**
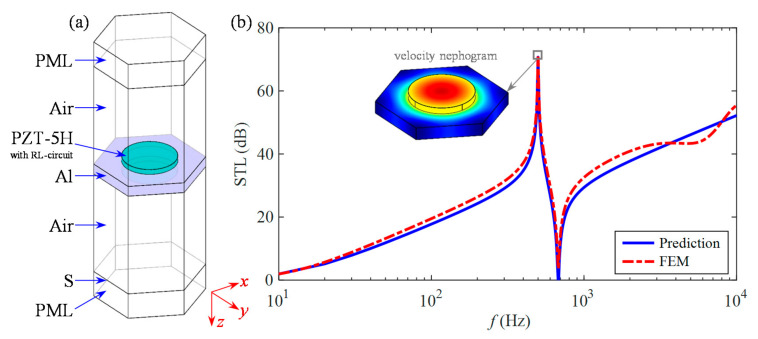
Finite-element simulation verification of the present metamaterial: (**a**) multiphysics geometry model and (**b**) sound insulation.

Moreover, based on the three modules of acoustics, structural mechanics, and AD/DC in COMSOL Multiphysics, an acoustic-structure fully coupled finite-element model of this metamaterial in an air environment is established as displayed in Figure 3a. Among the details of the model processing are the following: (1) simulating two semi-infinite spaces at the incident and transmitted ends by the perfectly matched layer (PML). (2) Defining the electropolar surface of one piezoceramic patch near the substrate as the ground, while the other is the terminal. (3) Adopting Floquet periodicity to approximate the infinite metamaterial structure. A plane wave impinges on the upper surface of the present metamaterial with only a single resonant frequency *f*_r_ = 500 Hz in the direction of elevation angle *θ* = 0° and azimuth angle *φ* = 0°, and the resulting global STL is illustrated in the pair of theoretical prediction and simulation analysis in Figure 3b. Among them, each piezoceramic has an external RL shunt circuit with *R* = 100 Ω and *L* = 4 H.

In Figure 3b, both STL curves show an overall frequency-dependent increase, with a pair of isolated peaks and valleys, which have high consistency and coincidence in the computational frequency band from 10 Hz to 10 kHz. With the help of the metamaterial velocity nephogram at the peak frequency, it can be learned that the sound insulation peak is attributed to the piezoelectric resonator formed by the piezoceramics flexibly connected to the substrate in a single unit cell.

## 4. Results and Discussion

This section successively reveals the sound insulation mechanisms and investigates the change law of dual equivalent properties, to delve into the high-performance broadband STL in the low-mid frequency band of this metamaterial with multi-modal resonant energy-dissipation modes.

### 4.1. Sound Insulation Mechanisms

**Figure 4:**
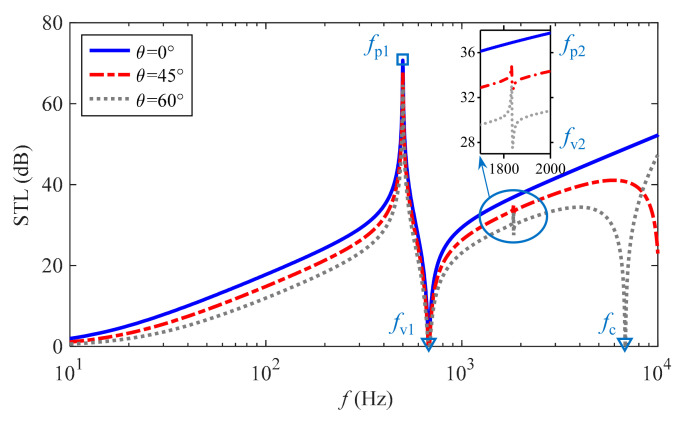
Sound insulation properties of the metamaterial with single-modal resonant frequency vary with elevation angle.

For a periodic metamaterial similar to the one depicted in Figure 1, the sound insulation performance is not affected by the azimuth angle *φ*. Therefore, the STL of this metamaterial thin-plate described in Section 3 above is explored under the incident conditions of a plane wave in the direction *φ* = 0° and *θ* = (0°, 45°, 60°), and the variation pattern is shown in Figure 4.

It is evident that a new pair of isolated peaks and valleys are grown with increasing elevation angle, and the 3rd trough frequency *f*_c_ moves toward the low-frequency direction. On the whole, the sound insulation of the present metamaterial decreases with the elevation angle increases, and only the amount of STL in the region between 1st crest frequency *f*_p1_ and 1st trough frequency *f*_v1_ is less affected.

**Figure 5:**
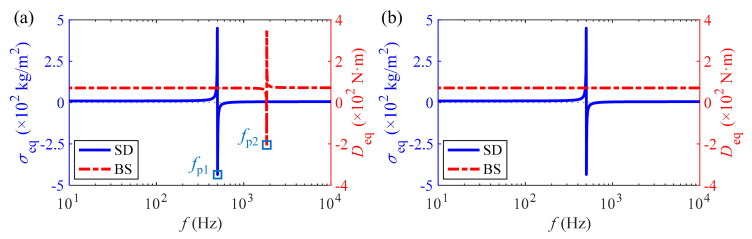
Effect of the RL shunt circuit with *R* = 100 Ω on dual equivalent properties of the metamaterial: (**a**) *L* = 4 H and (**b**) *L* = 0 H.

With respect to the 1st and 2nd sound insulation peaks, the corresponding generation mechanisms are different, which is reflected in the dual equivalent properties of the metamaterial as demonstrated in Figure 5a. In combination with Figure 3b, the former is due to the excellent characteristics of each mass-spring system consisting of piezoceramics flexibly connected to the substrate for vibration absorbing and energy conversion, which is manifested as the equivalent negative SD with the largest amplitude at the frequency point *f*_p1_. The latter is attributed to the largest amplitude of the equivalent negative BS at frequency *f*_p2_, however, the essential cause is the resonant dissipation of shunt circuits generated by the internal capacitance $C_{p}^{T}$ of piezoceramics and the inductance *L* of the corresponding shunt circuits. In particular, the equivalent negative BS characteristic of the metamaterial can be found to disappear by comparing and analyzing the *D*_eq_ curves in Figure 5a,b when the inductance *L* in RL-circuits changes from 4 H to 0 H.

It is worth noting that there is a rather peculiar phenomenon in Figure 4, that is, the STL curve with *θ* = 0° has no 2nd crest and 2nd trough, which is due to the normal incidence of a plane wave when the sound insulation properties of the metamaterial are controlled only by the equivalent mass (namely SD), while the equivalent BS performs practically no function. Quantitatively, the mechanical impedance of the metamaterial in Equation (28) can be rewritten as follows:

(33) $$Z_{M}=D_{\mathrm{eq}}{\left(k_{0}\sin\theta \right)}^{4}-\sigma_{\mathrm{eq}}\omega^{2}$$

Then, *Z*_M_ = −σ_eq_*ω*^2^ when *θ* = 0°, and the equivalent BS of the metamaterial has no effect.

**Figure 6:**
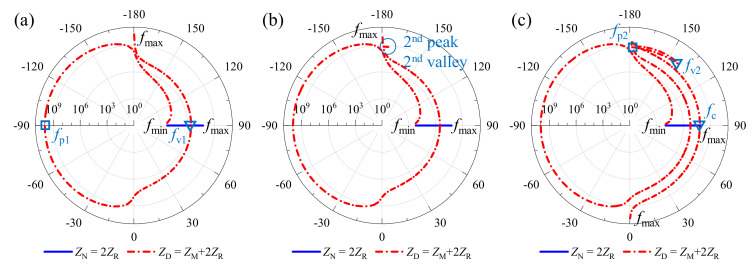
Impedance properties of the metamaterial vary with elevation angle: (**a**) *θ* = 0°; (**b**) *θ* = 45°; and (**c**) *θ* = 60°.

Meanwhile, by dissecting the impedance characteristics of the metamaterial when a plane wave incident at different elevation angle *θ* = (0°, 45°, 60°) as displayed in Figure 6, the mechanism of the three sound insulation valleys can be revealed while deepening the energy-dissipation principle of the two STL peaks in Figure 4. By comparing Figure 6a–c, it can be observed that the amplitude of *Z*_N_ increases with frequency and the phase remains constant, while both of *Z*_D_ change dynamically. Simultaneously, compared with the two *Z*_D_ curves with *θ* = 45° and *θ* = 60°, the impedance *Z*_D_ of *θ* = 0° has a smooth global transition and lacks two mutations in amplitude and phase, which correspond to the 2nd peak and 2nd valley, respectively. Taking the phase of *Z*_N_ as a reference, the transmission coefficient magnitude in Equation (30) decreases and the STL increases when the phase of *Z*_D_ is in the diffusion state, and vice versa. Furthermore, with the help of Figure 6, it can be revealed that the 1st peak frequency *f*_p1_ of the STL curves in Figure 4 appears at the opposite phase position of *Z*_D_ and *Z*_N_, and then the 1st valley frequency *f*_v1_ and 3rd trough frequency (coincident frequency) *f*_c_ correspond to the intersection of *Z*_D_ and *Z*_N_ with the same amplitude and phase in turn. Here, there is *Z*_M_ = 0 Pa·s/m, that is

(34) $$D_{\mathrm{eq}}{\left(k_{0}\sin\theta \right)}^{4}-\sigma_{\mathrm{eq}}\omega^{2}=0$$

and the valley frequencies *f*_v1_ and *f*_c_ are as follows:

(35) $$f_{v1}=\left\{\begin{array}{ll} f_{r}\sqrt{\left(1+\chi \frac{2\rho_{p}h_{p}}{\sigma_{s}}\right)\left(1+i\eta_{r}\right)}, & \left(\theta =0^{{}^{\circ}}\right)\\ \min\left(\frac{c_{0}^{2}}{2\pi \sin^{2}\theta }\sqrt{\frac{\sigma_{\mathrm{eq}}}{D_{\mathrm{eq}}}}\right), & \left(\theta \neq 0^{{}^{\circ}}\right) \end{array}\right.$$

(36) $$f_{c}=\max\left(\frac{c_{0}^{2}}{2\pi \sin^{2}\theta }\sqrt{\frac{\sigma_{\mathrm{eq}}}{D_{\mathrm{eq}}}}\right)$$

It is worth noting that the coincidence frequency *f*_c_ → ∞ of the metamaterial in the plane wave normal incidence, namely, no coincidence phenomenon occurs.

### 4.2. Change Law of Double Negative Characteristics

In view of the aforementioned correspondence between the two STL peak frequencies and the frequency points at the maximum amplitude of double equivalent negative characteristics. To this end, this section focuses on exploring the influence of the geometric and material parameters of this metamaterial on the equivalent negative SD and BS, namely the soundproof peak frequencies.

#### 4.2.1. Impact of Geometric Parameters

**Figure 7:**
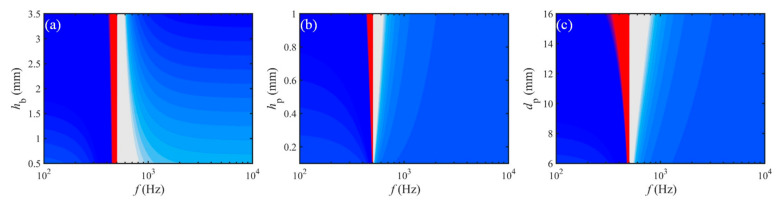
Variation of the equivalent SD with geometric parameters: (**a**) *h*_b_, (**b**) *h*_p_, and (**c**) *d*_p_.

The dual equivalent properties of the present metamaterial vary with variable parameters in the frequency domain, which can be fully illustrated by top-view 2D nephograms with the color transition from white via blue to red, indicating the change from the minimum to maximum amplitude values. In particular, the variation of equivalent SD with geometric parameters is depicted in Figure 7. It can be learned that the three parameters *h*_b_, *h*_p_, and *d*_p_ have no effect on the 1st peak frequency *f*_p1_, but only play a role in the negative characteristic bandwidth (subsequently abbreviated NCB) of *σ*_eq_. Among them, the NCB decreases with increasing *h*_b_, and varies inversely with *h*_p_ and *d*_p_. Then, in the corresponding STL curve, the 1st valley frequency *f*_v1_ shifts to the low-frequency direction with increasing *h*_b_, and to the opposite high-frequency direction with growing *h*_p_ and *d*_p_.

**Figure 8:**
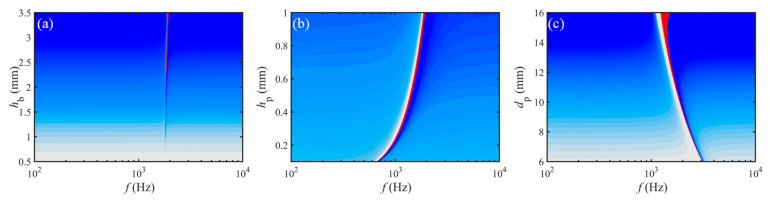
Change of equivalent BS with geometric properties: (**a**) *h*_b_, (**b**) *h*_p_, and (**c**) *d*_p_.

However, the change of equivalent BS with the above three geometric parameters is shown in Figure 8, and the *D*_eq_ is more complexly affected compared to the homologous *σ*_eq_. First of all, the equivalent BS for all three increases significantly with the variable. Secondly, the 2nd crest frequency, *f*_p2_, increases with increasing *h*_b_ and *h*_p_, but the sensitivity of *h*_p_ is higher; while diminishes with enhancing *d*_p_. That is, the *f*_p2_ in the corresponding STL curve shifts in the high-frequency direction with magnifying *h*_b_ and *h*_p_ but moves in the opposite direction with the amplification of *d*_p_. Finally, the NCB is not markedly influenced by *h*_b_ and *h*_p_ and improves with raising *d*_p_ by inches.

#### 4.2.2. Influence of Material Parameters

**Figure 9:**
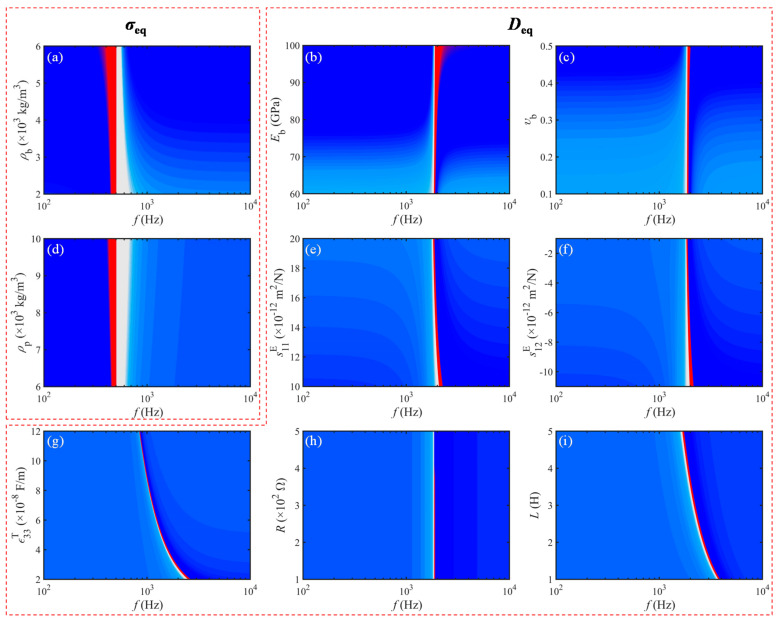
Dual equivalent properties vary with material parameters: (**a**) *ρ*_b_, (**b**) *E*_b_, (**c**) *υ*_b_, (**d**) *ρ*_p_, (**e**) $s_{11}^{E}$, (**f**) $s_{12}^{E}$, (**g**) $\varepsilon_{33}^{T}$, (**h**) *R*, and (**i**) *L*.

Mentioning the relationship between the dual equivalent properties and material characteristics in the present metamaterial, in combination with Equations (17) and (18), it is learned that the equivalent SD is only related to *ρ*_b_ and *ρ*_p_, while the equivalent BS is affected by seven material parameters, including *E*_b_, *υ*_b_, $s_{11}^{E}$, $s_{12}^{E}$, $\varepsilon_{33}^{T}$, *R*, and *L*. In the meantime, the variation of the double equivalent *σ*_eq_ and *D*_eq_ with all material parameters is illustrated in Figure 9.

From the 2D nephograms (a) and (d) of *σ*_eq_ in Figure 9, it can be seen that the 1st peak frequency *f*_p1_ corresponding to the maximum amplitude of the equivalent negative SD of the present metamaterial is fixed and independent of the density properties. The NCBs show a tendency to shrink and broaden with increasing *ρ*_b_ and *ρ*_p_, respectively. However, the remaining nephograms on the variation of *D*_eq_ with seven material parameters have more complicated rules. Firstly, for the effect of the material parameters *E*_b_ and *υ*_b_ of the substrate, the *D*_eq_ changes as displayed in the subgraphs (b) and (c) of Figure 9. The amplitude of *D*_eq_ amplifies for both, while the NCB and 2nd crest frequency *f*_p2_ at the maximum value of equivalent negative BS are less affected. Then, as the elastic and dielectric constants of the piezoceramics increase, the amplitude values of *D*_eq_ show a trend of enhancing and flattening, respectively, and the *f*_p2_ and NCB become progressively smaller, as depicted in Figure 9e–g. Last but not least, the RL-circuit parameters function only in the extremum region of equivalent BS. Among them, increasing the resistance *R* and inductance L, respectively, can successively suppress the magnitude of *D*_eq_ and modulate the resonant frequency of the circuits to move in the low-frequency direction, as shown in Figure 9h,i.

### 4.3. Low-Mid Frequency Broadband Transmission Loss

**Figure 10:**
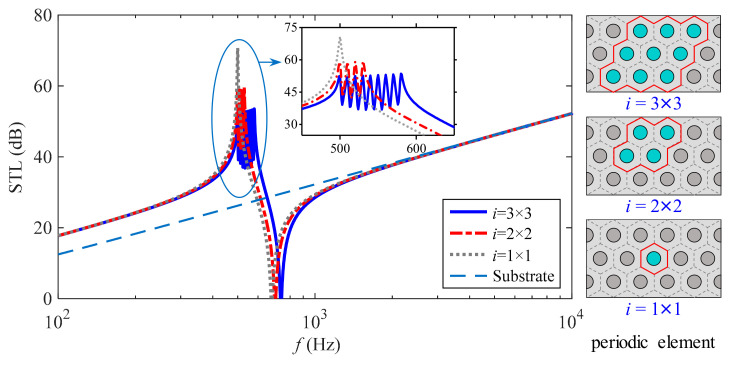
High-performance broadband sound insulation of the present metamaterial.

Based on the aforementioned analysis of the sound insulation mechanisms and change law of dual equivalent properties of the present metamaterial, the broadening potential of distributed multi-modal resonant energy-dissipation modes on the high-performance STL bandwidth in the low-mid frequency range is explored. For this purpose, taking the metamaterial with subwavelength piezoelectric arrays in Figure 1 as the prototype, three metamaterials containing *i* = (3 × 3, 2 × 2, 1 × 1) unit cell(s) in one single periodic element are designed, respectively, and their theoretical predictions under the condition of plane wave normal incidence are compared with the STL of the substrate as demonstrated in Figure 10. In particular, the resonant frequency of the mass-spring system in *i*-th unit cell of one single periodic element is *f*_r,*i*_ = *f*_r_ + 10 (*i* − 1) Hz.

As can be seen from Figure 10, the resonant frequencies of a distributed mass-spring system in a single periodic element affect only the first pair of insulation crest and trough, while as the number of multi-modal frequencies increases, the first peak of this metamaterial changes from one to a cluster, and peak amplitude decreases. There is a unique frequency *f*_s_ between the multi-modal frequencies and 1st valley frequency *f*_v1_ of the STL curve for the corresponding metamaterial, where the metamaterial and substrate are satisfied with equal amounts of sound insulation, then the implicit expression of *f*_s_ is

(37) $$f_{s}=\left\{\begin{array}{ll} f=\sqrt{-\frac{\chi \rho_{p}h_{p}}{ab\sigma_{s}}\sum_{i=1}^{ab}{\left(\frac{1}{f^{2}}-\frac{1}{f_{r,i}^{2}\left(1+i\eta_{r,i}\right)}\right)}^{-1}}, & \left(\theta =0^{{}^{\circ}}\right)\\ f=\frac{c_{0}^{2}}{2\pi \sin^{2}\theta }\sqrt{\frac{\sigma_{\mathrm{eq}}-\sigma_{s}}{D_{\mathrm{eq}}-D_{s}}}, & \left(\theta \neq 0^{{}^{\circ}}\right) \end{array}\right.$$

Moreover, the STL curves of the three metamaterials can be divided into three stages compared to the sound insulation properties of the substrate, namely, an improved region with a calculation frequency below *f*_s_, a poor region near the 1st valley frequency *f*_v1_, and a high-frequency coincidence region (Note: There is no coincidence phenomenon in the plane-wave normal incidence condition). In the improved region, the sound insulation of all three metamaterials is more than 5 dB above that of the substrate, even up to 44.49 dB.

With the STL of 20 dB as a reference value, the three metamaterials are able to exceed the lower frequency limit of 244 Hz for the substrate to meet this requirement and move the frequency point to 129 Hz, which is an extension of 115 Hz in the low-frequency direction. Meanwhile, in the frequency range of the multi-modal peak frequency and the 1st valley frequency *f*_v1_, the frequency limits for the three metamaterials with *i* = (1 × 1, 2 × 2, 3 × 3) to achieve this requirement are 633 Hz, 654 Hz and 693 Hz, respectively, and the two kinds of metamaterials with *i* = (2 × 2, 3 × 3) broaden 21 Hz and 60 Hz in the high-frequency direction compared to the metamaterial with *i* = 1 × 1.

In summary, the present metamaterial containing distributed piezoelectric resonators has significant sound insulation enhancement in the improved region and is coupled with multi-modal resonant energy-dissipation modes that broaden the upper-frequency limit of this region, which has excellent sound insulation potential in low-mid frequency broadband or specific frequency bands.

## 5. Conclusions

This paper proposes an acoustic metamaterial with distributed piezoelectric vibrators and develops a complete acoustic prediction model based on the effective medium (EM) method and classical plate theory (CPT), focusing on equivalent negative characteristics and sound insulations. Moreover, with the aid of the acoustic-structure fully coupled finite-element model built by COMSOL Multiphysics, the correctness of the theoretical model is verified via STL simulations. After that, the sound insulation mechanisms, characteristic change, and low-mid frequency broadband STL of the present metamaterial are investigated in-depth, and three main conclusions are drawn as follows:

(1). The present metamaterial is an excellent sound insulation metamaterial, which possesses dual equivalent negative properties, that is, equivalent negative surface density (SD) and equivalent negative bending stiffness (BS).
(2). The sound insulation peaks correspond to equivalent negative SD and BS, respectively, which are attributed to the distributed piezoelectric resonators and resonant shunts in turn, and the multi-modal resonant energy-dissipation modes effectively broaden the upper-frequency limit of the improved region.
(3). The resistive and inductive elements in an external RL shunt circuit serve in turn to suppress the amplitude of the equivalent negative bending stiffness (BS) for the present metamaterial and to modulate the resonant frequency of this circuit, similar to the damping and spring elements in a mechanical system, respectively.
(4). The low-mid frequency sound insulation of this metamaterial is better than that of the substrate over 5 dB, even up to 44.49 dB.

## Data Availability

The data used to support the findings of this study are included within the article.
